# Development and validation of a novel prognostic score for HBV-related acute-on-chronic liver failure

**DOI:** 10.3389/fmed.2026.1837035

**Published:** 2026-07-17

**Authors:** Qian Song, Lin Lv, Chuanfei Li, Zhechuan Mei, Shengtao Liao

**Affiliations:** 1 Department of Gastroenterology, The Second Affiliated Hospital of Chongqing Medical University, Chongqing, China; 2 Chongqing Municipal Health Commission Key Laboratory of Precision Diagnosis and Treatment of Liver Cirrhosis and Complications, The Second Affiliated Hospital of Chongqing Medical University, Chongqing, China

**Keywords:** HBV-ACLF, MELD score, model, prediction, prognosis

## Abstract

**Background:**

Acute-on-chronic liver failure patients have high short-term mortality rates, and HBV infection is the most common cause. Current prognostic models for HBV-ACLF patients have limitations. Thus, the development of an ideal model remains an important unsolved problem.

**Aims:**

To develop a new model and validate its prognostic predictive capacity.

**Methods:**

Among 5854 liver failure patients admitted to the Second Affiliated Hospital of Chongqing Medical University from 2008 to 2023, 1170 HBV-ACLF cases were finally enrolled, which were randomly divided into a development cohort (*n* = 822) or validation cohort (*n* = 348). First, we obtained important predictive factors about 28-day and 90-day mortality using cox regression analysis and developed new prognostic scores. Second, the new scores were compared with current models through ROC curves, calibration curves and DCA curves. Third, using survival analysis, the new scores were risk-stratified according to optimal cut-off values.

**Results:**

The AHF-MELD and AHN-MELD scores’ predictive capacity were stronger than MELD, MELD-sodium, CLIF-C OF and AARC scores and comparable to CLIF-C ACLF, COSSH ACLF and COSSH ACLF II scores. There was no significant difference between the observed and the predicted 28-day and 90-day mortality probabilities of new scores. The AHF-MELD and AHN-MELD scores have positive net benefits over a wide range of threshold probabilities and outperform conventional scores. The 28-day and 90-day survival rates were significantly lower in high-risk groups than in low-risk groups.

**Conclusion:**

The AHF-MELD and AHN-MELD scores may serve as potential predictive tools for the short-term prognosis of HBV-ACLF patients. Our study may provide useful information for clinicians managing these patients.

## Introduction

Acute-on-chronic liver failure is an acute exacerbation occurring on the basis of cirrhosis (with or without decompensation). It is a complication distinct from acute decompensated cirrhosis, accompanied by multi-organ failure (liver, kidney, brain, coagulation function, respiratory and circulatory systems), and is characterized by a high short-term mortality rate (28-day mortality ≥ 15%) (1–3). Notably, HBV infection remains an important cause of cirrhosis in Asian countries, and as a result, HBV- ACLF accounts for 15% of Asia-Pacific ACLF cases (4). Thus, early evaluation of prognosis may prevent bad outcomes.

Although, several prognostic models, including MELD, MELD-sodium (5), AARC (6), CLIF-C OF (7), CLIF-C ACLF (8), COSSH-ACLF and COSSH-ACLF II (9), have been established and updated for ACLF by researchers domestically and internationally, a key improvement is still needed. Among above scores, only the study population of COSSH-ACLF and COSSH-ACLF II scores are patients with chronic hepatitis B. Their formulas remain relatively complex for routine bedside use. Therefore, the development of a simpler and more objective prognostic model for HBV-ACLF remains an important unsolved problem.

The MELD score is widely used in various liver diseases to predict clinical prognosis, which derives the MELD-Na and iMELD scores. It includes 3 indicators of serum bilirubin, creatinine (Scr) and INR. Moreover, Growing evidences have shown (9–12) that age, HE, serum ferritin, and neutrophil count are important factors in predicting the prognosis of ACLF patients. Accordingly, we hypothesize that combining these indicators with MELD may perform well in prognostic prediction for patients with HBV-ACLF. To test the hypothesis, we analyzed the clinical data of 1170 cases to validate the predictive performance of the new scores.

## Patients and methods

### Study design

**FIGURE 1:**
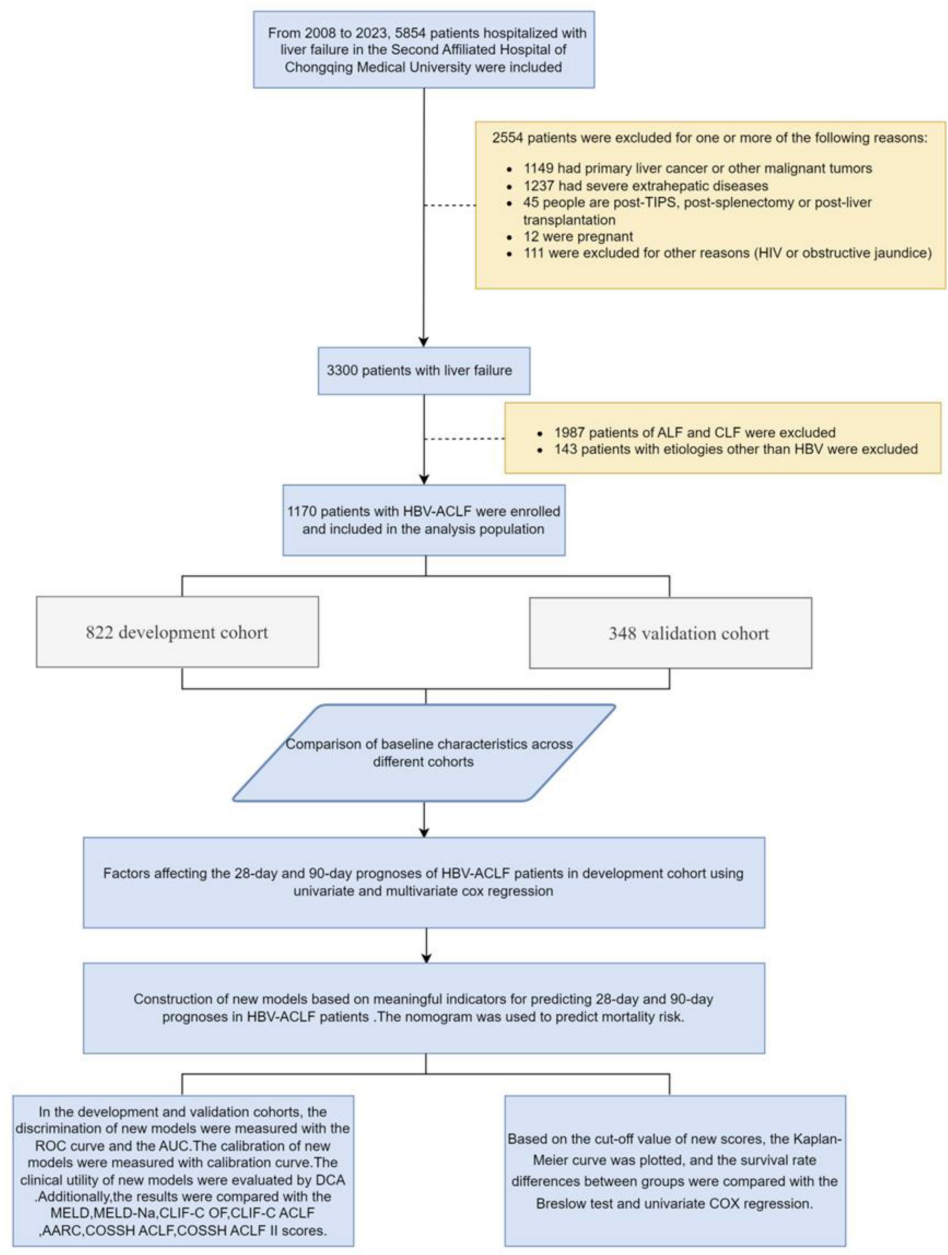
The flowchart of the study design.

Among 5854 liver failure patients admitted to the Second Affiliated Hospital of Chongqing Medical University from 2008 to 2023, 1170 HBV-ACLF cases were finally included according to the inclusion and exclusion criteria. First, they were randomly assigned to development cohort (*n* = 822) or validation cohort (*n* = 348). Second, patients in the development cohort were assigned to death or survival group according to 28-day and 90-day prognosis. Furthermore, we obtained the important predictive factors about 28-day and 90-day mortality and developed new prognostic scores. Third, the predictive power of the new score was validated in the validation cohort. Fourth, the new score was risk stratified to accurately identify those at high risk of death. The flowchart of the study design is described in Figure 1.

### Patients

Patients were eligible for inclusion if they met the 2024 COSSH-ACLF standards during hospitalization: jaundice (serum bilirubin ≥ 12 mg/dL) and coagulopathy (INR ≥ 1.5). Key exclusion criteria were malignant tumors such as primary liver cancer; serious extrahepatic diseases; postsurgical status of liver transplantation, splenectomy, or TIPS; pregnant, obstructive jaundice or HIV-positive persons; non-HBV etiology. This protocol was approved by the Ethics Committee of the Second Affiliated Hospital of Chongqing Medical University, number: 141-1/2023.

### Procedures

First, general condition, laboratory indicators, and complications in 24 h of the HBV-ACLF diagnosis at our institution were harvested, and MELD, MELD-Na, AARC, CLIF-C OF, CLIF-C ACLF, COSSH-ACLF and COSSH-ACLF II scores were calculated. Second, patients were followed until they died, or day 90, whichever came first. In addition, standardized medical therapy was given to all included patients. In this study, all enrolled HBV-ACLF patients had positive serum HBV DNA or HBeAg. Those who met the criteria for antiviral therapy were treated with nucleoside analogs or interferon.

### Statistical analysis

Random forest imputation was used to handle randomly missing data. The quantitative data were described by the means and standard deviations (SDs) or medians and interquartile range. Student’s *t*-test or Wilcoxon Mann-Whitney test was used to determine the statistical significance of differences between two groups. The categorical data was analyzed with the use of the chi-square test. First, we used cox regression analysis to identify the important factors about 28-day and 90-day prognosis in the development cohort. Accordingly, new prognostic scores for HBV-ACLF patients were developed with the use of the cox regression and described with the use of nomograms. Second, the discrimination, calibration and clinical utility of the new scores were assessed by using the ROC curve, the calibration curve and the DCA curve, respectively, in the development cohort. Comparison of the AUCs was performed with the use of the DeLong test. Furthermore, the same method was applied in the validation cohort to assess predictive capacity. Third, based on the optimal cut-off value of the new score, survival analysis was performed for risk stratification. Univariate Cox regression was used to compare the 28-day and 90-day survival rates between groups, and the HR (95% CI) and *p*-value were calculated.

## Results

### Baseline characteristics

Baseline characteristics of the 1170 HBV-ACLF patients were equally distributed between the development and validation cohorts (Table 1). We observed no statistically significant difference in the general conditions (gender, age, history of diabetes or cirrhosis) between groups (*P* > 0.05). The incidence of complications (SBP, HE, ascites, HRS, GI bleeding and SIRS) did not differ statistically between groups (*P* > 0.05), and the most common complications were SBP (627[76.3%] patients in the development cohort vs. 257[73.9%]in the validation cohort) and ascites (411 [50.0%]vs. 163[46.8%]). All laboratory indicators, including hemoglobin, leukocyte count, neutrophil count, neutrophil percentage, lymphocyte percentage, monocyte count, platelet count, CRP, PCT, ferritin, albumin, ALT, AST, GGT, lactate, INR, PTA, PT, fibrinogen, D-dimer, creatinine, urea nitrogen, sodium, HBeAg positivity, HBV-DNA quantification, alpha fetoprotein, and ammonia were not statistically different between groups at baseline (*P* > 0.05) other than bilirubin and ALP. There was no statistical difference in scoring systems (MELD, MELD-Na, CLIF-C OF, CLIF-C ACLF, COSSH-ACLF II) (*P* > 0.05) other than AARC and COSSH-ACLF.

**TABLE 1:** Baseline characteristics of hepatitis B virus-related acute-on-chronic liver failure in the development and validation cohorts.

Characteristic	Development cohort (*n* = 822)	Validation cohort (*n* = 348)	*p*
Male, *n* (%)	139 (16.9)	55 (15.8)	0.705
Age (years)	47.45 (12.75)	47.45 (12.10)	0.997
Diabetic, *n* (%)	73 (8.9)	35 (10.1)	0.599
Cirrhosis, *n* (%)	569 (69.2)	236 (67.8)	0.685
Liver transplant, *n* (%)	6 (0.7)	0 (0.0)	0.25
Complications, n (%)			
SBP	627 (76.3)	257 (73.9)	0.419
HE	259 (31.5)	127 (36.5)	0.112
Ascites	411 (50.0)	163 (46.8)	0.355
HRS	107 (13.0)	54 (15.5)	0.297
GI bleeding	96 (11.7)	51 (14.7)	0.191
SIRS	118 (14.4)	54 (15.5)	0.672
Laboratory data			
Hemoglobin (g/L)	125.00 [107.00, 138.00]	125.00 [111.00, 137.25]	0.449
Leukocyte count (×10^9^/L)	7.14 [5.23, 9.34]	7.40 [5.43, 10.17]	0.198
Neutrophil count (×10^9^/L)	5.30 [3.76, 7.31]	5.60 [3.69, 7.94]	0.247
Neutrophil percentage (%)	74.50 (9.61)	74.71 (9.53)	0.732
Lymphocyte percentage (%)	14.51 [9.60, 20.50]	13.60 [9.90, 19.50]	0.476
Monocyte count (×10^9^/L)	0.58 [0.43, 0.80]	0.62 [0.42, 0.90]	0.101
Platelet count (×10^9^/L)	97.00 [64.00, 132.00]	97.00 [61.00, 140.00]	0.438
CRP (mg/L)	11.48 [7.06, 20.12]	12.09 [7.70, 19.63]	0.616
PCT (ng/mL)	0.74 [0.50, 1.17]	0.71 [0.46, 1.20]	0.352
Ferritin (μg/L)	1221.63 [919.27, 1500.00]	1208.15 [940.66, 1426.42]	0.792
Bilirubin (umol/L)	317.85 [258.87, 394.28]	332.66 [273.70, 415.80]	0.014
Albumin (g/L)	30.52 (4.29)	30.68 (4.03)	0.547
ALT (U/L)	293.75 [112.00, 760.50]	317.65 [109.60, 776.25]	0.806
AST (U/L)	220.95 [120.00, 563.90]	244.64 [123.47, 569.83]	0.366
GGT (U/L)	82.90 [52.30, 132.75]	77.05 [53.98, 122.00]	0.498
ALP (U/L)	146.00 [119.00, 181.75]	152.50 [125.75, 189.25]	0.033
Lactate (mmol/L)	3.20 [2.55, 4.10]	3.20 [2.50, 3.98]	0.525
INR	2.36 [1.89, 3.07]	2.40 [1.93, 3.21]	0.214
PTA (%)	32.00 [24.00, 41.00]	32.00 [23.00, 40.25]	0.235
PT (S)	25.70 [21.70, 31.70]	26.20 [21.90, 32.80]	0.236
Fibrinogen (g/L)	1.46 [1.16, 1.77]	1.47 [1.12, 1.76]	0.96
D-dimer (ng/mL)	1100.00 [553.60, 1786.29]	1115.86 [583.23, 1713.95]	0.929
Creatinine (umol/L)	64.25 [53.82, 84.97]	64.95 [53.60, 84.53]	0.647
Urea nitrogen (mmol/L)	4.45 [3.25, 6.79]	4.29 [3.19, 7.13]	0.721
Sodium (mmol/L)	135.36 [132.18, 138.09]	135.39 [132.06, 138.60]	0.901
HBeAg positive, *n* (%)	264 (32.1)	117 (33.6)	0.665
HBV-DNA (log_10_IU/mL)	5.16 [3.38, 6.67]	5.23 [3.36, 6.80]	0.85
Alpha fetoprotein (ug/L)	75.09 [25.25, 199.85]	67.93 [19.50, 171.52]	0.126
Ammonia (umol/L)	47.10 [30.85, 70.00]	47.41 [32.00, 72.00]	0.77
Scores			
MELD	24.30 [20.83, 29.54]	25.02 [20.65, 29.95]	0.305
MELD-sodium	26.05 [22.14, 33.23]	26.88 [22.29, 34.68]	0.286
CLIF-C OF	10.00 [9.00, 11.00]	11.00 [9.00, 12.00]	0.264
CLIF-C ACLF	45.99 (8.38)	46.52 (7.83)	0.315
AARC	10.00 [9.00, 11.00]	10.00 [9.00, 11.00]	0.038
COSSH ACLF	6.80 [6.18, 7.77]	7.01 [6.28, 8.10]	0.039
COSSH ACLF II	7.51 [6.82, 8.35]	7.67 [6.85, 8.53]	0.065

### Development of new prognostic scores

**FIGURE 2:**
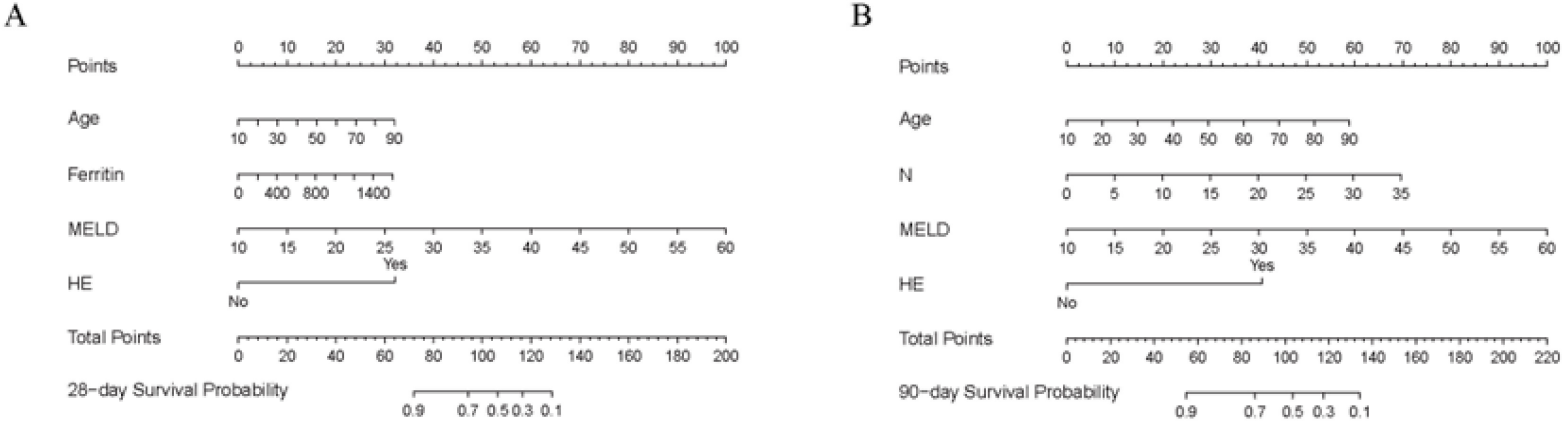
Nomogram for predicting 28-day and 90-day mortality of HBV-ACLF patients in development cohort. **(A)** 28-day; **(B)** 90-day.

First, backward stepwise regression analysis indicated that age, HE, serum ferritin and MELD score independently influenced the 28-day prognosis of HBV-ACLF patients; age, HE, neutrophil count and MELD score influenced the 90-day prognosis in the development cohort (*P* < 0.05) (Table 2). Second, we developed a new score using age, HE, serum ferritin and MELD score (AHF-MELD) to predict the 28-day probability of death (Figure 2A) in HBV-ACLF patients. We developed a new score using age, HE, neutrophil count and MELD (AHN-MELD) score to predict the 90-day probability of death (Figure 2B).

**TABLE 2:** Cox regression analyses for 28-day and 90-day mortality of HBV-ACLF patients in the development cohort.

Characteristic	28 d				90 d			
	Univariate analysis		Multivariate analysis		Univariate analysis		Multivariate analysis	
	HR (95% Cl)	*p*-value	HR (95% Cl)	*p*-value	HR (95% Cl)	*p*-value	HR (95% Cl)	*p*-value
Male, *n* (%)	1.03 [0.64, 1.68]	0.89			0.91 [0.61, 1.34]	0.618		
Age (years)	1.05 [1.03, 1.06]	<0.001	1.03 [1.01, 1.04]	0.001	1.05 [1.04, 1.06]	<0.001	1.03 [1.02, 1.04]	<0.001
Diabetic, *n* (%)	1.42 [0.76, 2.65]	0.267			1.49 [0.87, 2.53]	0.143		
Cirrhosis, *n* (%)	0.84 [0.58, 1.23]	0.377			1.06 [0.76, 1.48]	0.727		
Complications, n (%)								
SBP	2.43 [1.43, 4.12]	0.001			2.48 [1.59, 3.85]	<0.001		
HE	11.28 [7.27, 17.51]	<0.001	5.33 [3.07–9.25]	<0.001	7.90 [5.66, 11.02]	<0.001	4.12 [2.83, 5.99]	<0.001
Ascites	2.67 [1.79, 3.97]	<0.001			2.79 [1.99, 3.90]	<0.001		
HRS	4.12 [2.70, 6.28]	<0.001			4.26 [2.95, 6.15]	<0.001		
GI bleeding	3.13 [2.00, 4.91]	<0.001			3.05 [2.06, 4.52]	<0.001		
SIRS	2.96 [1.92, 4.56]	<0.001			2.78 [1.90, 4.08]	<0.001		
Laboratory data								
Hemoglobin (g/L)	1.00 [0.99, 1.00]	0.231			0.99 [0.98, 1.00]	0.007		
Leukocyte count (×10^9^/L)	1.10 [1.07, 1.13]	<0.001			1.09 [1.06, 1.12]	<0.001		
Neutrophil count (×10^9^/L)	1.11 [1.08, 1.15]	<0.001			1.11 [1.08, 1.14]	<0.001	1.09 [1.06, 1.13]	<0.001
Neutrophil percentage (%)	1.06 [1.04, 1.09]	<0.001			1.07 [1.05, 1.08]	<0.001		
Lymphocyte percentage (%)	0.92 [0.89, 0.95]	<0.001			0.91 [0.89, 0.94]	<0.001		
Monocyte count (×10^9^/L)	2.85 [1.79, 4.55]	<0.001			2.61 [1.72, 3.97]	<0.001		
Platelet count (×10^9^/L)	1.00 [0.99, 1.00]	0.107			0.99 [0.99, 1.00]	0.002		
CRP (mg/L)	1.01 [1.00, 1.02]	0.006			1.01 [1.01, 1.02]	<0.001		
PCT (ng/mL)	1.06 [1.03, 1.10]	<0.001			1.08 [1.05, 1.10]	<0.001		
Ferritin (μg/L)	1.00 [1.00, 1.00]	0.001	1.00 [1.00–1.00]	<0.001	1.00 [1.00, 1.00]	0.052		
Bilirubin (umol/L)	1.00 [1.00, 1.00]	<0.001			1.01 [1.00, 1.01]	<0.001		
Albumin (g/L)	0.95 [0.91, 1.00]	0.029			0.95 [0.92, 0.99]	0.008		
ALT (U/L)	1.00 [1.00, 1.00]	0.721			1.00 [1.00, 1.00]	0.166		
AST (U/L)	1.00 [1.00, 1.00]	0.182			1.00 [1.00, 1.00]	0.6		
GGT (U/L)	1.00 [1.00, 1.00]	0.097			1.00 [1.00, 1.00]	0.119		
ALP (U/L)	1.00 [0.99, 1.00]	0.183			1.00 [0.99, 1.00]	0.048		
Lactate (mmol/L)	1.28 [1.21, 1.35]	<0.001			1.24 [1.18, 1.30]	<0.001		
INR	1.38 [1.30, 1.45]	<0.001			1.37 [1.30, 1.44]	<0.001		
PTA (%)	0.90 [0.88, 0.92]	<0.001			0.92 [0.90, 0.93]	<0.001		
PT (S)	1.06 [1.05, 1.07]	<0.001			1.05 [1.05, 1.06]	<0.001		
Fibrinogen (g/L)	0.32 [0.21, 0.49]	<0.001			0.39 [0.28, 0.56]	<0.001		
D-dimer (ng/mL)	1.00 [1.00, 1.00]	<0.001			1.00 [1.00, 1.00]	<0.001		
Creatinine (umol/L)	1.00 [1.00, 1.00]	<0.001			1.00 [1.00, 1.00]	<0.001		
Urea nitrogen (mmol/L)	1.11 [1.08, 1.13]	<0.001			1.11 [1.08, 1.13]	<0.001		
Sodium (mmol/L)	0.96 [0.92, 0.99]	0.007			0.94 [0.92, 0.97]	<0.001		
HBeAg positive, *n* (%)	0.72 [0.48, 1.09]	0.119			0.78 [0.56, 1.10]	0.158		
HBV-DNA (log_10_IU/mL)	1.00 [1.00, 1.00]	<0.001			1.00 [1.00, 1.00]	0.003		
Alpha fetoprotein (ug/L)	1.00 [1.00, 1.00]	<0.001			1.00 [1.00, 1.00]	<0.001		
Ammonia (umol/L)	1.01 [1.01, 1.02]	<0.001			1.01 [1.01, 1.01]	<0.001		
MELD	1.16 [1.13, 1.18]	<0.001	1.11 [1.07–1.16]	<0.001	1.14 [1.12, 1.16]	<0.001	1.09 [1.05, 1.13]	<0.001

### Discrimination of new scores

**FIGURE 3:**
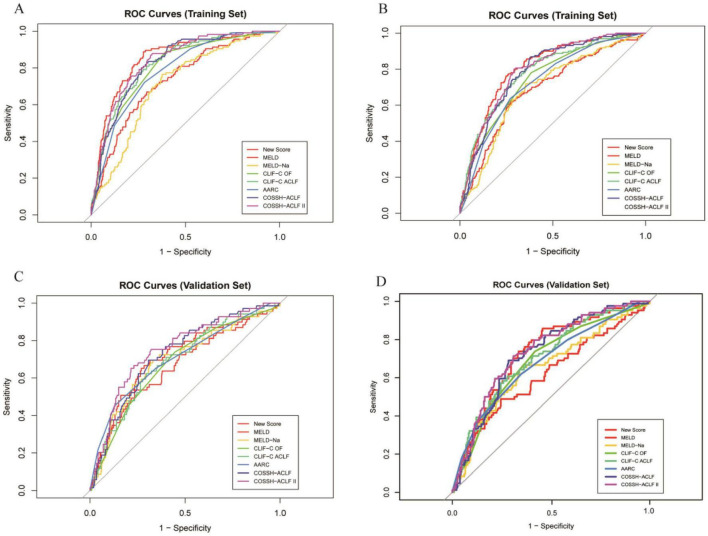
ROC curves comparison of new score and 7 other prognostic models in predicting 28-day and 90-day mortality of HBV-ACLF patients. **(A,B)** 28-day and 90-day in development cohort; **(C,D)** 28-day and 90-day in validation cohort.

For predicting 28-day mortality of HBV-ACLF patients in the development cohort, the new score exhibited 0.851AUC, 89% sensitivity and 72% specificity. The AUCs were provided as follows: New Score > COSSH-ACLF II > COSSH-ACLF > CLIF-C ACLF > CLIF-C OF > AARC > MELD > MELD-Na. Furthermore, delong test showed that the differences between the new score and the CLIF-C ACLF, COSSH ACLF and COSSH ACLF II scores were not statistically significant (*P* > 0.05). However, the differences between the new score and the MELD, MELD-sodium, CLIF-C OF and AARC scores were statistically significant (*P* < 0.05) (Figure 3A and Supplementary Table 1).

For predicting 90-day mortality, the new score exhibited 0.809AUC, 76% sensitivity and 75% specificity. The AUCs were as follows: New Score > COSSH-ACLF II > COSSH-ACLF > CLIF-C ACLF > CLIF-C OF > AARC > MELD-Na > MELD. Additionally, we found that the Delong test’s results in 28-day and 90-day model were similar (Figure 3B and Supplementary Table 1).

### Validation of new scores

For predicting 28-day mortality of HBV-ACLF patients in the validation cohort, the new score had 0.71AUC, 65% sensitivity and 73% specificity. The AUCs were listed as follows: COSSH-ACLF II > COSSH-ACLF > New Score > AARC > CLIF-C ACLF > MELD-Na > CLIF-C OF > MELD. Delong test indicated that the differences between the new score and the MELD, MELD-sodium, CLIF-C OF, CLIF-C ACLF, AARC, COSSH ACLF scores were not statistically significant (*P* > 0.05). However, the differences between the new score and the COSSH ACLF II score were statistically significant (*P* < 0.05) (Figure 3C and Supplementary Table 2).

For predicting 90-day mortality, the new score had 0.73AUC, 74% sensitivity and 66% specificity. The AUCs were as follows: COSSH-ACLF II > New Score > COSSH-ACLF > CLIF-C ACLF > CLIF-C OF > AARC > MELD-Na > MELD. Furthermore, delong test demonstrated that the differences between the new score and the CLIF-C ACLF, CLIF-C OF, COSSH ACLF and COSSH ACLF II scores were not statistically significant (*P* > 0.05). But, the differences between the new score and the MELD-sodium, MELD and AARC scores were statistically significant (*P* < 0.05) (Figure 3D and Supplementary Table 2).

### Calibration of new scores

**FIGURE 4:**
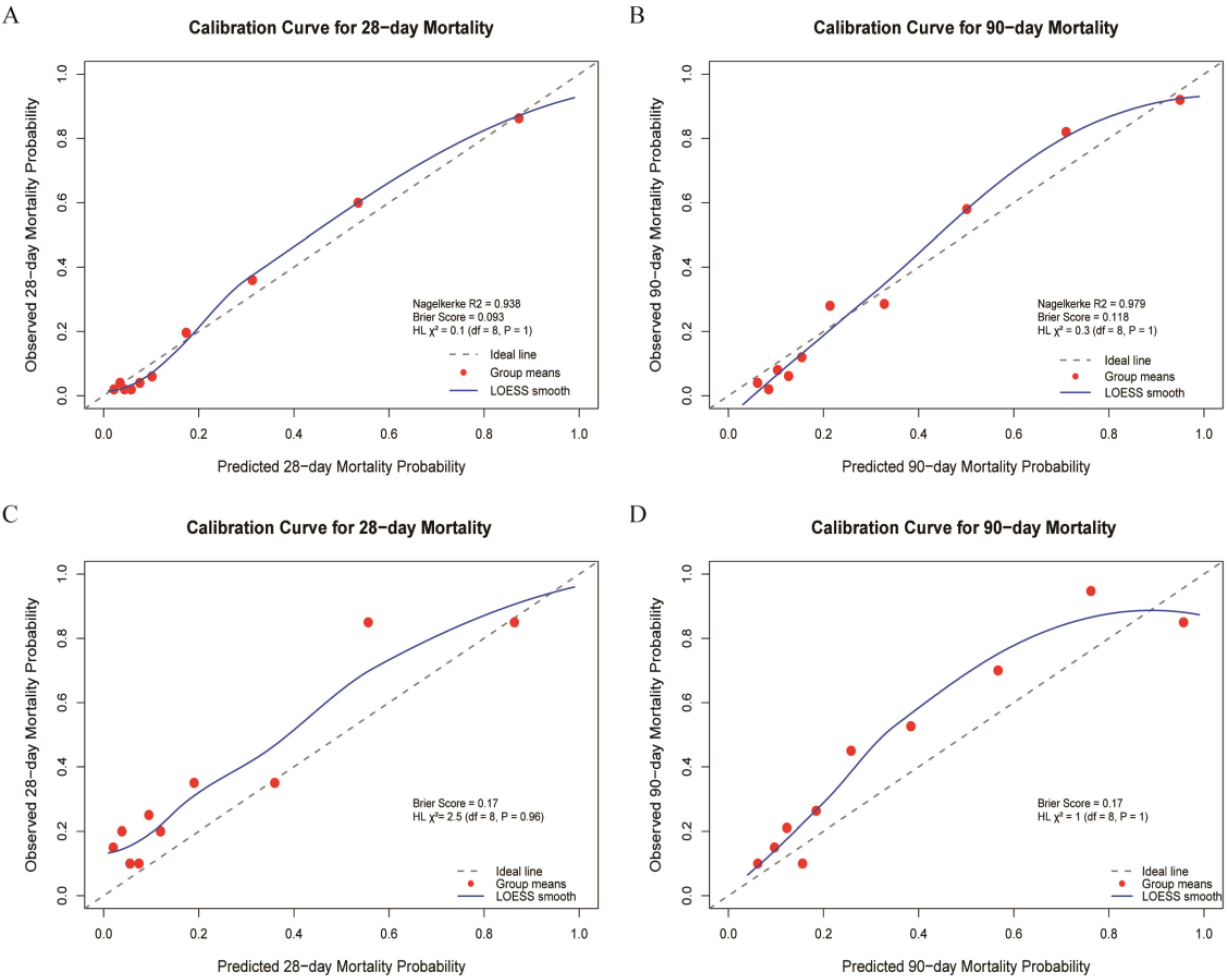
Calibration curves of new score for predicting 28-day and 90-day mortality of HBV-ACLF patients. **(A,B)** 28-day and 90-day in development cohort; **(C,D)** 28-day and 90-day in validation cohort. R^2^ and the Brier score: a higher R^2^ value and a lower Brier score indicated better performance.

Calibration assessment showed that in the development cohort, for 28-day prediction: HL test *P* = 1, Brier = 0.093, R^2^ = 0.938 (Figure 4A); for 90-day prediction: HL test *P* = 1, Brier = 0.118, R^2^ = 0.979 (Figure 4B). In the validation cohort, for 28-day prediction: HL test *P* = 0.96, Brier = 0.17, (Figure 4C); for 90-day prediction: HL test *P* = 1, Brier = 0.17, (Figure 4D). All metrics indicated no significant deviation between predicted and observed probabilities, confirming good calibration.

### Clinical utility of new scores

**FIGURE 5:**
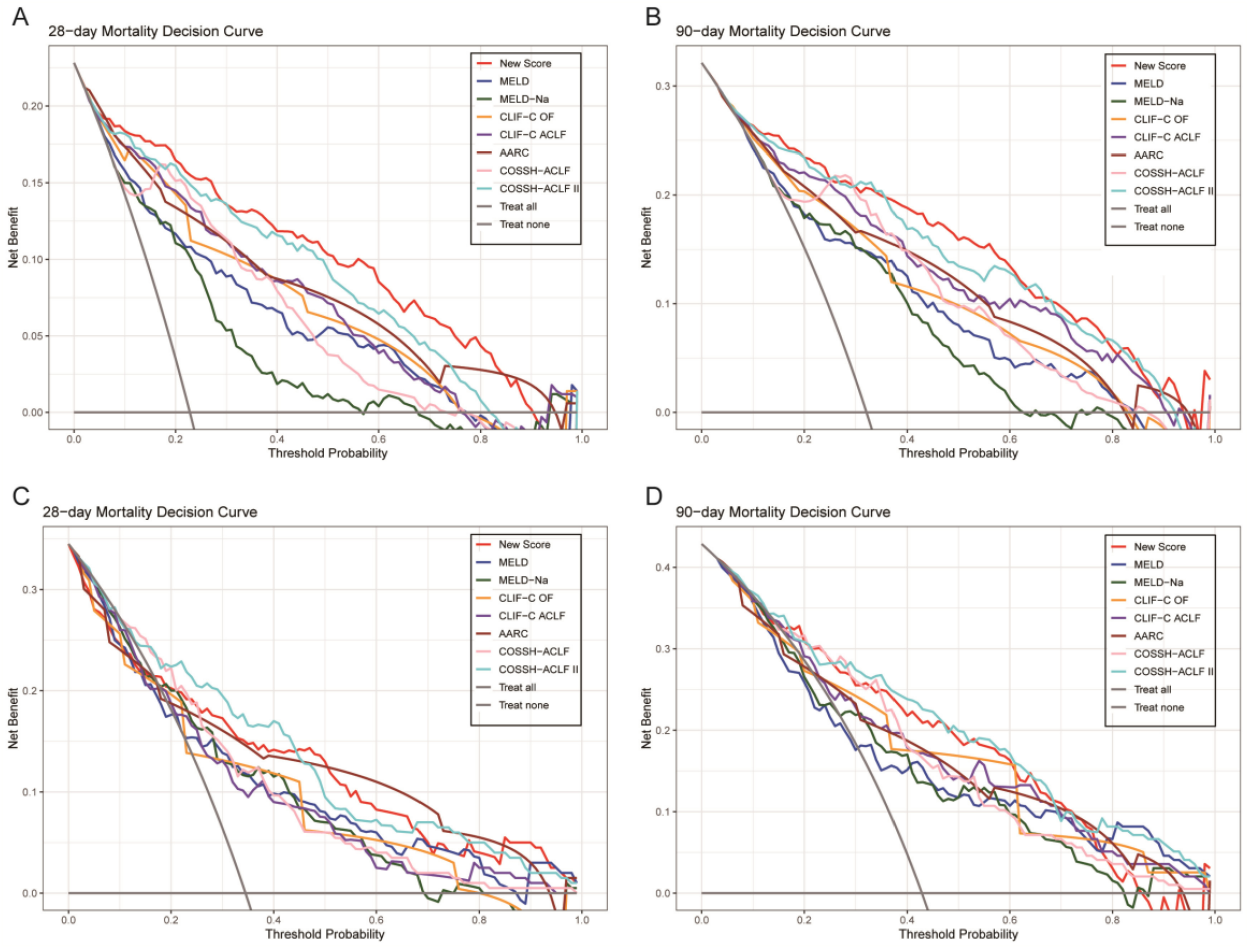
DCA curves of new score for predicting 28-day and 90-day mortality of HBV-ACLF patients. **(A,B)** 28-day and 90-day in development cohort; **(C,D)** 28-day and 90-day in validation cohort.

Decision curve analysis (DCA) showed that, in both the development and validation cohorts and for both 28-day and 90-day endpoints, the new score provided positive net benefit over a wide range of threshold probabilities. Compared with conventional scores such as MELD, MELD-sodium, CLIF-C OF, CLIF-C ACLF, AARC, COSSH ACLF, the new score had higher net benefit; compared with existing models such as COSSH-ACLF II, the new score had comparable or slightly superior net benefit. These results indicate that the new score has good clinical utility (Figure 5).

### Risk stratification

**FIGURE 6:**
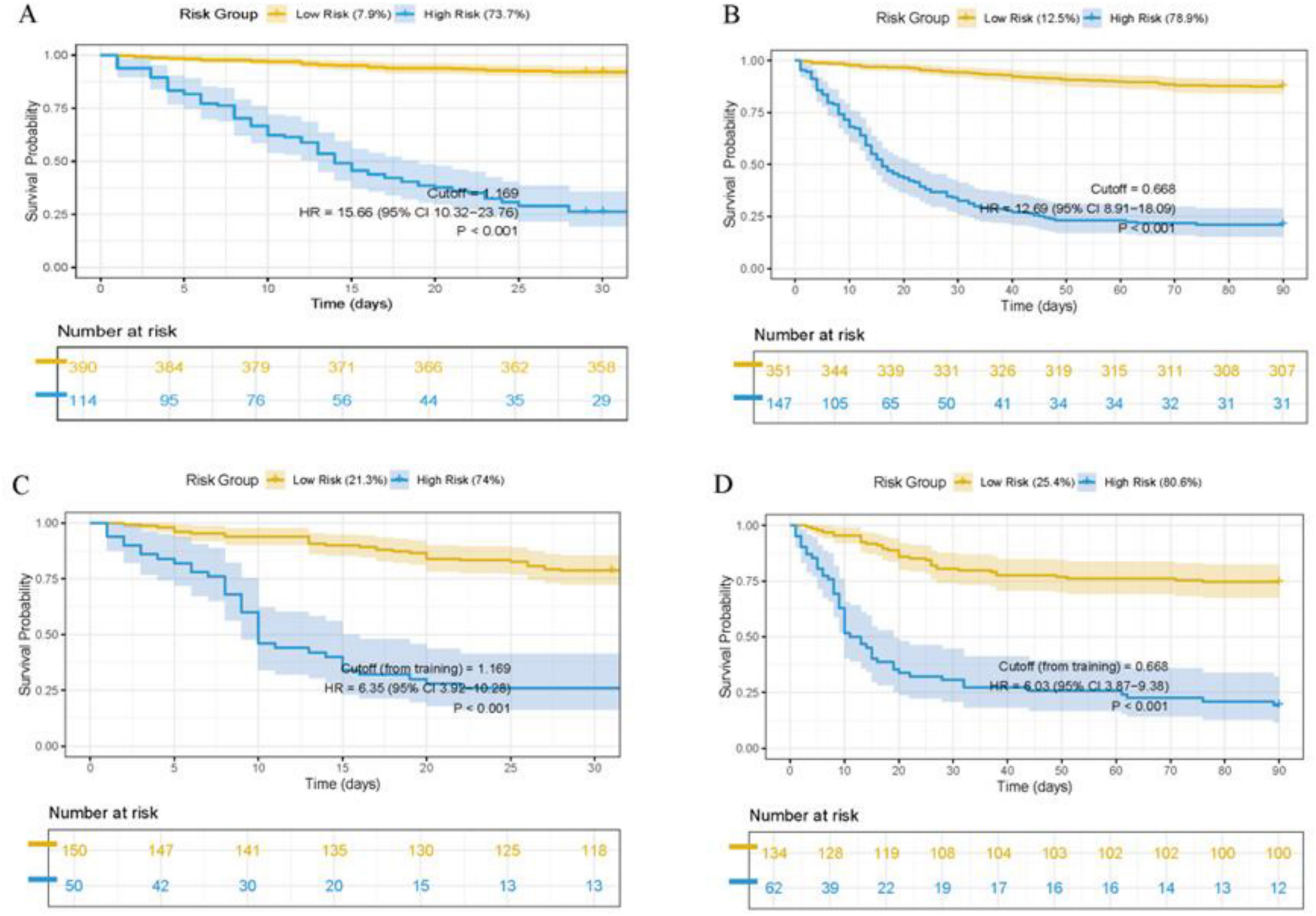
Risk stratification of new score. **(A,B)** Development cohort; **(C,D)** Validation cohort.

We divided the new score into high-risk and low-risk groups based on the optimal cut-off value. In the development cohort, the high-risk group had significantly higher risk of death at 28 days (HR = 15.66, 95% CI [10.32–23.76], *P* < 0.001) and 90 days (HR = 12.69, 95% CI [8.91–18.09], *P* < 0.001) than the low-risk group (Figures 6A, B). Moreover, the results observed in the validation cohort were consistent with those in the development cohort (28-day: HR = 6.35, 95% CI [3.92–10.28], *P* < 0.001; 90-day: HR = 6.03, 95% CI [3.87–9.38], *P* < 0.001, Figures 6C, D).

## Discussion

In this study, we observed that the baseline characteristics were equally distributed between cohorts. Using cox regression, we found that: (i) age, HE, serum ferritin and MELD score independently influenced the 28-day prognosis of HBV-ACLF patients (ii) age, HE, neutrophil and MELD score independently influenced the 90-day prognosis of HBV-ACLF patients. On this basis, we utilized age, HE, serum ferritin, neutrophil and MELD score to develop new scores: AHF-MELD score (28 d), AHN-MELD score (90 d), and graphed nomograms to display the 28-day and 90-day risk of death. In addition, we examined the predictive capacity of this new score. First, in the development cohort, the new score had the highest AUC for predicting 28-day and 90-day mortality in HBV-ACLF patients. Further delong test proved that the differences between the new score and these scores (CLIF-C OF, MELD and MELD-sodium, AARC) were statistically significant (*P* < 0.05) besides CLIF-C ACLF, COSSH ACLF and COSSH ACLF II scores. Second, in the validation cohort, for predicting 28-day mortality, the AUC of the new score was only lower than that of the COSSH-ACLF and COSSH-ACLF II scores. Except for the COSSH-ACLF II score, the differences between the new score and the other scores (MELD, MELD-sodium, CLIF-C OF, CLIF-C ACLF, AARC, COSSH-ACLF) were not statistically significant (*P* > 0.05). For predicting 90-day mortality, the AUC of the new score was only lower than that of the COSSH-ACLF II score. Except for the MELD, MELD-sodium, and AARC scores, the differences between the new score and the other scores (CLIF-C OF, CLIF-C ACLF, COSSH-ACLF, COSSH-ACLF II) were not statistically significant (*P* > 0.05). Third, from the calibration curves, we found no significant difference between the observed and the predicted 28-day and 90-day mortality probabilities of the new score. Finally, cox analysis demonstrated that all high-risk groups had significantly lower 28-day and 90-day survival rates than those of the low-risk groups, according to the cut-off point. These results support the hypothesis that the new score probably performed well in prognostic prediction for HBV-ACLF patients.

Most attention has been focused on age, HE, ferritin, neutrophil, and MELD indicators. First, age is an independent risk factor for the prognosis of liver failure patients. Both previous studies and our evidence demonstrate that older age is associated with higher mortality risk (13, 14). Second, HE is a serious complication of HBV-ACLF; its occurrence is closely related to elevated blood ammonia, cerebral edema, and systemic inflammation, significantly increasing short-term mortality (15). Third, ferritin, an acute-phase reactant, reflects the degree of hepatocyte necrosis and systemic inflammatory status; hyperferritinemia is associated with multi-organ failure and poor prognosis in ACLF patients (16). Fourth, an elevated neutrophil count indicates systemic inflammation, and its level is positively correlated with infection, sepsis, and the severity of extrahepatic organ injury, making it an important predictor of prognosis in HBV-ACLF (17). Fifth, since its establishment in 2000, the MELD score has been widely used for prognostic assessment of liver disease (18), but it still has limitations — serum creatinine cannot be solely attributed to hepatic factors, and its predictive performance for HBV-ACLF remains suboptimal (19). Numerous studies have indicated (20, 21) that modified MELD-based scores have better prognostic capability. Based on the above analysis, we combined age, HE, ferritin, neutrophil count, and the MELD score to further improve the predictive accuracy for 28-day and 90-day mortality in HBV-ACLF patients.

Our study has several strengths. First, the new scores are founded on simple and objective indicators. Second, the large sample size of the development and validation cohorts provided sufficient power for the analyses. Third, the new score outperforms or non-inferior to existing HBV-ACLF prognostic scores. In the future, we will conduct a prospective cohort study to identify the significance of this new score in clinical practice.

In conclusion, we suggest that the AHF-MELD and AHN-MELD scores may serve as potential predictive models for 28-day and 90-day prognosis in HBV-ACLF patients. Our study may provide useful information for clinicians managing these patients. Future studies are needed to externally validate our findings and further clarify the potential role of these scores in clinical practice.

## Data Availability

The original contributions presented in this study are included in this article/Supplementary material, further inquiries can be directed to the corresponding authors.
