# Supplementary material for: Development and validation of a novel prognostic score for HBV-related acute-on-chronic liver failure
Source: Front Med (Lausanne). 2026 Jul 17;13:1837035. doi: 10.3389/fmed.2026.1837035 (PMC13425134; doi:10.3389/fmed.2026.1837035)
Supplement: Supplementary file 1 [file Table_1.docx]

**Supplemental Table 1 Discrimination ability of new score in development cohort**

| score | 28d | | | | | 90d | | | | |
| --- | --- | --- | --- | --- | --- | --- | --- | --- | --- | --- |
|  | AUC | Cut-off | Sensitivity | Specificity | p value^✻^ | AUC | Cut-off | Sensitivity | Specificity | p value^✻^ |
| New score | 0.851 | 0.45 | 0.89 | 0.72 |  | 0.809 | 0.46 | 0.76 | 0.75 |  |
| MELD | 0.734 | 26.66 | 0.67 | 0.70 | <0.001 | 0.69 | 26.66 | 0.62 | 0.73 | <0.001 |
| MELD-sodium | 0.709 | 27.25 | 0.77 | 0.62 | <0.001 | 0.692 | 27.25 | 0.72 | 0.65 | <0.001 |
| CLIF-C OF | 0.806 | 10.50 | 0.89 | 0.61 | 0.003 | 0.751 | 10.50 | 0.78 | 0.62 | <0.001 |
| CLIF-C ACLF | 0.826 | 48.75 | 0.79 | 0.72 | 0.18 | 0.785 | 48.98 | 0.70 | 0.75 | 0.079 |
| AARC | 0.785 | 10.50 | 0.72 | 0.72 | <0.001 | 0.73 | 10.50 | 0.64 | 0.73 | <0.001 |
| COSSH ACLF | 0.827 | 7.33 | 0.80 | 0.73 | 0.089 | 0.787 | 6.87 | 0.86 | 0.62 | 0.07 |
| COSSH ACLF II | 0.84 | 7.80 | 0.87 | 0.68 | 0.462 | 0.798 | 7.80 | 0.80 | 0.71 | 0.259 |

^✻^DeLong test

| score | 28d | | | | | 90d | | | | |
| --- | --- | --- | --- | --- | --- | --- | --- | --- | --- | --- |
|  | AUC | Cut-off | Sensitivity | Specificity | p value^✻^ | AUC | Cut-off | Sensitivity | Specificity | p value^✻^ |
| New score | 0.71 | 0.64 | 0.65 | 0.73 |  | 0.73 | 0.14 | 0.74 | 0.66 |  |
| MELD | 0.661 | 28.48 | 0.54 | 0.77 | 0.110 | 0.617 | 28.48 | 0.49 | 0.75 | ＜0.001 |
| MELD-sodium | 0.692 | 28.68 | 0.70 | 0.67 | 0.632 | 0.652 | 28.68 | 0.64 | 0.66 | 0.023 |
| CLIF-C OF | 0.679 | 10.50 | 0.74 | 0.55 | 0.230 | 0.69 | 10.50 | 0.74 | 0.58 | 0.110 |
| CLIF-C ACLF | 0.693 | 48.45 | 0.62 | 0.70 | 0.515 | 0.706 | 48.45 | 0.61 | 0.72 | 0.228 |
| AARC | 0.705 | 11.50 | 0.45 | 0.87 | 0.850 | 0.681 | 10.50 | 0.62 | 0.65 | 0.046 |
| COSSH ACLF | 0.728 | 7.30 | 0.70 | 0.69 | 0.374 | 0.724 | 7.30 | 0.69 | 0.72 | 0.785 |
| COSSH ACLF II | 0.753 | 8.26 | 0.65 | 0.78 | 0.025 | 0.738 | 7.88 | 0.71 | 0.69 | 0.596 |

**Supplemental Table 2 Discrimination ability of new score in validation cohort**

^✻^DeLong test
